# The Genome of *Undifilum oxytropis* Provides Insights into Swainsonine Biosynthesis and Locoism

**DOI:** 10.1038/srep30760

**Published:** 2016-08-01

**Authors:** Hao Lu, Haiyun Quan, Zhenhui Ren, Shuai Wang, Ruixu Xue, Baoyu Zhao

**Affiliations:** 1 College of Veterinary Medicine, Northwest A&F University, Yangling, Shaanxi, 712100, China

## Abstract

*Undifilum oxytropis* is a fungal endophyte of locoweeds. It produces swainsonine, which is the principal toxic ingredient of locoweeds. However, the genes, pathways and mechanisms of swainsonine biosynthesis are not known. In this study, the genome of *U. oxytropis* was firstly sequenced and assembled into a 70.05 megabases (Mb) draft genome, which encoded 11,057 protein-coding genes, and 54% of them were similar to current publicly available sequences. *U. oxytropis* genes were annotated and 164 putative genes were annotated into enzymes, such as Saccharopine dehydrogenase, Saccharopine oxidase, and Pyrroline-5-carboxylate reductase, hypothesized to be involved in the biosynthesis pathway of swainsonine. The genome sequence and gene annotation of *U. oxytropis* will provide new insights into functional analyses. The characterization of genes in swainsonine biosynthesis will greatly facilitate locoweed poisoning research and help direct locoism management.

Locoweeds are poisonous legumes that belong to the genera *Oxytropis* and *Astragalus*. After ingesting these toxic plants, livestock can develop diseases characterised by chronic dysfunction of the nervous system^1^,^2^,^3^. In China, the morbidity and death rate of livestock caused by locoweed poisoning have increased annually and led to large economic losses in the western rangelands of China in the last fifty years^4^.

The principal toxic ingredient of locoweeds is the indolizidine alkaloid swainsonine (SW)^5^,^6^,^7^,^8^,^9^,^10^. SW primarily inhibits the activities of Golgi apparatus α- mannosidase II (MAN2A1) and lysosome α- mannosidase (MAN2B1)^11^,^12^,^13^. Recent studies showed that SW treatment could reduce the expression of MAN2A1 and MAN2B1 in some animal tissues and cells^14^,^15^,^16^.

Swainsonine was shown to be produced by two unrelated fungi, *Rhizoctonia leguminicola* and *Metarhizium anisopliae*^17^,^18^. In 2009, a fungal endophyte, *Undifilum oxytropis*^19^, was reported to produce swainsonine in locoweeds^20^. The *Undifilum* is now categorized as *Alternaria* sect. *Undifilum*^21^,^22^, which belong to *Pleosporaceae* in the phylum *Ascomycota. Undifilum* species have been found to be associated with swainsonine-containing *Astragalus* and *Oxytropis* species in China and North America^19^,^23^,^24^. In these plants, swainsonine levels were correlated positively with *U. oxytropis* content^25^, which provided original evidence that fungal genetics might play an important role in swainsonine production^20^. The metabolic pathway that leads to the formation of swainsonine has been studied in *Metarhizium*, and *R. leguminocola*^26^,^27^,^28^, in which the swainsonine metabolic pathway appears to be a branch of the lysine biodegradation pathway^26^.

However, the biosynthetic pathway of swainsonine in *U. oxytropis* is still not clear. With the development of next-generation DNA sequencing, annotation of the genome of *U. oxytropis* can provide important data on genes involved in swainsonine biosynthesis. In this study, we firstly sequenced and assembled the genome of *U. oxytropis* that produced swainsonine. We made full annotations with the predicted genes in this genome. With integrated gene prediction and annotation, we illuminated the biosynthesis and predicted key enzymes that may regulate SW in *U. oxytropis*. This study will further contribute to the understanding of the biosynthesis pathway and metabolic mechanism of swainsonine, and provide an important reference for prevention and treatment of locoweed poisoning in livestock.

## Results

### Genome sequencing and analysis

After filtering low quality and adapter contamination reads, the genome of *U. oxytropis* was sequenced by whole genome shotgun sequencing strategy and produced 3,950 Mb clean data (Table S1). The assembly was performed by SOAP *de novo* genome assembler^29^, which generated 9,757 contigs with N50 of 24,851 base pairs (bp) and then assembled into 6367 scaffolds with N50 of 33,191 bp. The lengths of scaffolds ranged from 1,000 bp to 345,265 bp (Table 1). Finally, we assembled a 70.05 Mb draft genome sequence for *U. oxytropis*. The expected genome size was 79.71 Mb as determined by k-mer length (Figures S1, S2, S3), so these scaffolds covered 87.88% of the whole genome. The G + C content of the *U. oxytropis* genome was 40.37% (Figure S4). The features of the assembled genome sequences are shown in Table 1.

### The analysis of genome component

We identified 11,057 protein-coding genes with a total length of 18,400,467 bp, accounting for 26.27% of the genome by combining several different gene predictors, (Table S2). The gene number was equal to number of coding sequence (CDS), The total length of exons was also equal to total length of CDS (Table S2). The gene density was 157.8 genes/per Mbp and the average size of protein coding genes was 1,664.15 bp. Genes contained small exons (average 505.17 bp) and introns (average 149.64 bp). There were an average of 2.77 exons in one gene, which was similar to that found for other Ascomycetes^30^. *U. oxytropis* was predicted to have 11,057 protein coding genes, which was similar to the coding capacity of other Ascomycetes^30^. In addition, in the *U. oxytropis* genome, genes of <2000 bp account for 81%, gene length distribution 0 ~ 1000 bp account for 41.6% of the gene length, 1000 ~ 2000 bp account for 39.4% of the gene length, and >2000 bp account for 19% (Figure S5).

### Gene function annotation

**Figure 1:**
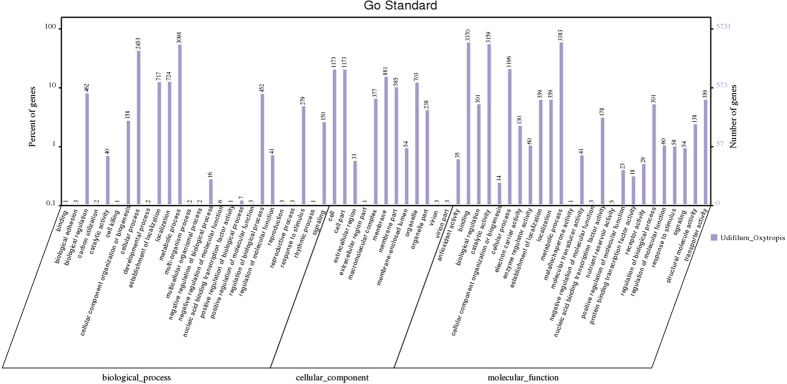
The GO function annotation of *U. oxytropis*. Distribution of genes in different GO function classification.

**Figure 2:**
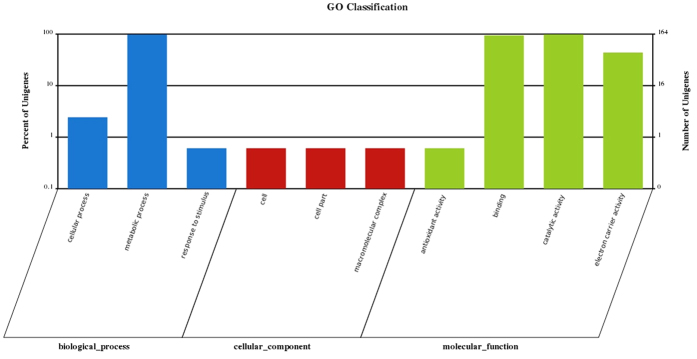
The GO function annotation screened 164 genes of *U. oxytropis*. Distribution screened 164 genes in different GO function classification.

**Figure 3:**
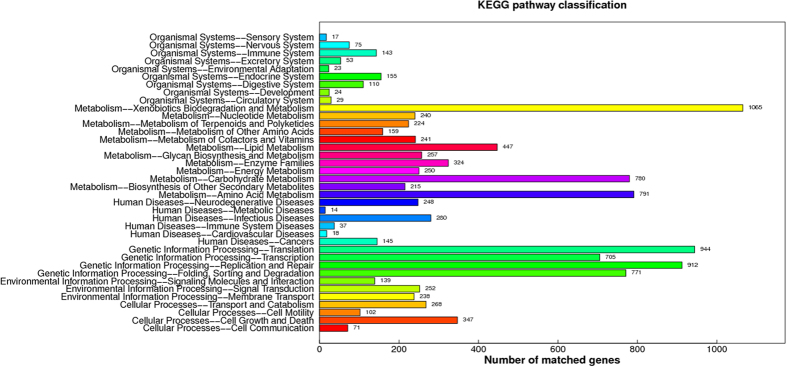
The KEGG function annotation in *U. oxytropis*. Distribution of genes in different KEGG categories.

We mapped our predicted proteins to Gene Ontology (GO) using homology search, 5,731 (51.8%) of which were assigned to GO terms, including 10,933, 2,354 and 5,470 genes that mapped to the molecular function, cellular component, and biological process categories, respectively. In molecular function, “metabolic process”, “binding” and “catalytic activity” were the top three categories, which matched 3,383, 3,370 and 3,159 genes, respectively. Within cellular component, “cell” and “cell part” were the top 2 categories, which matched 1,173 and 1,173 genes. In biological process, “metabolic process” and “cellular process” were the top 2 categories, which matched 3,098 and 2,453 genes (Fig. 1). Based on this, we screened 164 genes and assigned GO terms; the highest number of genes were found to be involved in metabolic process and catalytic activity (Fig. 2). Meanwhile, we also assigned 5,965 proteins according to the Kyoto Encyclopedia of Genes and Genomes (KEGG) database. The KEGG function classification is shown in Fig. 3, in which “Xenobiotics Biodegradation and Metabolism”, “Translation” and “replication and repair” were the top 3 categories, followed by “Amino Acid Metabolism” and “Carbohydrate Metabolism”, which included up to 1,065, 944, 912, 791 and 780 predicted *U. oxytropis* genes, respectively. As a result, about 54% of predicted genes were similar to sequences in public databases and only 5,092 genes were not similar to current public sequences, some of which might be *Undifilum* specific genes.

### The pathway of swainsonine synthesis

**Figure 4:**
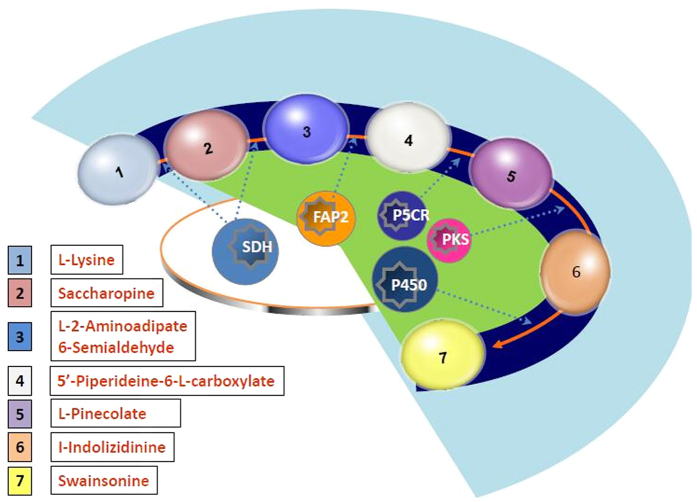
The putative swainsonine biosynthesis pathway in *U. oxytropis*. Enzymes involved in this pathway are: SDH: saccharopine dehydrogenase, [EC:1.5.1.7], K00290, [EC:1.5.1.9], K00292, [EC:1.5.1.10], K00293; FAP2: saccharopine oxidase, [EC: 1.5.3.1], K00301; P5CR: pyrroline-5-carboxylate reductase, [EC:1.5.1.2], K00286; PKS: polyketide synthase, [EC: 2.3.-.-]; P450: cytochrome P450, [EC: 1.14.-.-].

Swainsonine is of great importance in *U. oxytropis* because of its significant roles in immune regulation and anticancer activity^31^,^32^,^33^,^34^. Swainsonine can induce toxicosis in animals that consume the alkaloid. Therefore, it is very important to understand the biosynthesis of swainsonine and identify the key genes associated its biosynthesis. According to “Tropane, piperidine and pyridine alkaloid biosynthesis” pathway in KEGG (map00960), we speculated the putative swainsonine biosynthesis pathway in *U. oxytropis* (Fig. 4). We knew that swainsonine is a final product of L-lysine degradation, from L-Lysine to Saccharopine and L-2-aminoadipate 6-semialdehyde, the two intermediates can be obtained from L-lysine degradation (KEGG map00310), from the genome annotation results, and we found that 5 genes was annotated to a key enzyme, Saccharopine dehydrogenase (SDH, EC:1.5.1.-) of this pathway. In L-Lysine degradation process, from L-2-aminoadipate 6-semialdehyde to ∆^1^-piperideine-6-carboxylic acid (P6C), 1 gene was annotated to Saccharopine oxidase (FAP2, EC 1.5.3.1), furthermore, we found 2 genes annotated to pyrroline-5-carboxylate reductase (P5CR, EC:1.5.1.2) from P6C to L-pipecolate. However, we also found that many more genes that were annotated to two enzymes, polyketide synthase (PKS, EC: 2.3.-.-) and cytochrome P450 (P450, EC: 1.14.-.-), from L-pipecolate to swainsonine, the former was annotated to 79 genes, and the latter was 77 genes. To sum up, we found that some genes can be annotated to key enzymes, which were connected with swainsonine biosynthesis pathway. The important genes information annotated to some enzymes were shown in Table 2.

## Discussion

Swainsonine, as the active principle in locoweeds, is a water-soluble indole alkaloid and alpha-mannosidase inhibitor which blocks Golgi oligosaccharide processing. It has gained attention widely, because it represents a new class of compounds that could inhibit tumor growth and metastasis^34^. Swainsonine has also aroused interest because of its immunostimulatory properties and possible use in cancer chemotherapy^31^,^32^,^33^,^34^,^35^,^36^. However, swainsonine may have detrimental effects, especially on animals such as goats and sheep, which can be poisoned after ingesting swainsonine-containing locoweeds. Recent findings have indicated that swainsonine is produced by the slow growing endophytic fungus, *Undifilum oxytropis*^19^,^20^. However, little is known about the biosynthesis of swainsonine by *U. oxytropis.* In order to gain further understanding into the biosynthesis of swainsonine, genome sequencing and annotation of *U. oxytropis* are crucial. Here we sequenced the genome sequence of *U. oxytropis* by Solexa technology. We assembled the sequences into 6,367 scaffolds in 70.05 Mb sequences represented about 87.88% of the whole genome and annotated 11,057 gene models at genome level. We compared the genome sequencing and assemble of *U. oxytropis* with another swainsonine-producing fungus, *Metarhizium anisopliae*, and we found the genome of *U. oxytropis* is different from the genome of *M. anisopliae*, which is a small genome size (39.04 Mb), the higher G + C content (51.49%) and similar genes number (10,582 bp)^30^. *U. oxytropis* was also different from the related, but non-swainsonine produce *Altermaria arborescens*. The *Altermaria arborescens* genome had a genome size of 34 Mb, predicted 9,167 genes with an average length of 1.8 Kb and gene density of 3.7 Kb/gene and GC content of 51.34%^37^. Li *et al.* (2012) analyzed the proteome of *U. oxytropis* and categorized 52 proteins. Our results were similar to their findings. Within molecular function, our top two categories were binding, where Li *et al.* found 49% of the proteins were related to binding and catalytic activity, where 10% of the proteins were given that category by Li *et al.*^38^. In our work, within the biological process area, the largest number of genes were metabolic process which corresponds with 34% of the proteins within the biological process.

Currently, studies on swainsonine biosynthesis have mostly been performed on *Rhizoctonia leguminicola*, renamed as *Slafractonia leguminicola*^39^ and *Metarhizium anisopliae*^26^,^40^,^41^. However the detailed biosynthesis pathway is still unclear. The pathway for swainsonine biosynthesis has been partially characterized in *R. leguminicola* and *M. anisopliae*^26^. In the partially characterized pathway, L-pipecolic acid was shown to serve as a precursor for the production of swainsonine. In fungi like *Metarhizium* and *Rhizoctonia*, pipecolic acid is formed by the catabolism of L-lysine that in turn allows for the formation of the alkaloid, swainsonine^26^,^28^.

In the “Tropane, piperidine and pyridine alkaloid biosynthesis” pathway from KEGG map database (map00960), swainsonine is one of the final products in lysine metabolism. In the *Undifilum oxytropis* genome, we found some putative genes that were annotated to lysine degradation pathway. Thus, at the genome level, we confirmed that some key enzymes that could be involved in swainsonine biosynthesis are present in the fungus. Previous studies have established two basic routes for converting lysine into pipecolic acid, which are P2C pathway and P6C pathway^42^. The important intermediates are ∆^1^-piperideine-2-carboxylic acid (P2C) and ∆^1^-piperideine-6-carboxylic acid (P6C)^43^. The P6C pathway has been intensively studied in *R. leguminicola*, because the synthesis of pipecolic acid via this route represents the initial steps in the production of two toxic octahydroindolzine alkaloids, slaframine, and swainsonine^44^. Another experimental result implied that ∆^1^-piperideine-6-carboxylate, not ∆^1^-piperideine-2-carboxylate, was involved in the conversion of lysine to pipecolic acid, and this conclusion was fully supported by relevant proton NMR studies^27^. Subsequently, a chain of reactions have been established through which L-lysine was converted to saccharopine, which was in turn converted to P6C through oxidative cleavage. The latter was then readily reduced to pipecolic acid. A previously unrecognized flavin enzyme, Saccharopine oxidase was identified, which oxidatively cleaves saccharopine to yield P6C. Since saccharopine is a major metabolite in lysine degradation in *R. leguminicola*, saccharopine oxidase apparently functions to shunt saccharopine into secondary metabolism pathway to supply precursor (pipecolic acid) for slaframine and swainsonine production^28^. Several of these proteins were identified from proteomics work from *R. leguminicola*, including L-pipecolate oxidase, L-aminoadipate semialdehyde dehydrogenase, and saccharopine dehydrogenase. In addition, six cytochrome P450 and two PKS were identified^38^.

Fujii *et al.*^45^ found that *E. coli* pyrroline-5-carboxylate (P5C) reductase (EC 1.5.1.2) (encoded by proC gene) acted efficiently with *Flavobacterium lutescens* LAT to convert L-lysine into L-pipecolic acid^45^. It is noteworthy that P5C reductase is present in almost all organisms^46^. It is possible that in the microorganisms that produce L-pipecolic acid via P6C pathway, the universally conserved P5C reductase is actually responsible, at least in part, for the reduction of P6C into L-pipecolic acid^45^. Although little is known about the biosynthetic pathway of swainsonine in the endophytic fungus *Undifilum oxytropis*, genome sequencing and function annotation in this fungus will open avenues for future research on control of loco-disease. According to the putative swainsonine biosynthesis pathway in *U. oxytropis*, we have gained an insight into swainsonine metabolism pathway and some key enzymes involved in this process such as SDH, FAP2, P5CR, PKS and cytochromes P450 enzymes. These enzymes play a very important role in swainsonine biosynthesis. In our results, we found that many genes might be involved in swainsonine biosynthesis in *U. oxytropis*. After predicting some key enzymes of swainsonine biosynthesis pathway in *U. oxytropis*, 5 putative SDH genes, 1 putative FAP2 gene, 2 putative P5CR genes, 79 putative PKS genes and 77 putative cytochromes P450 genes were found.

Previous studies reported that P2C and P6C pathways are two basic routes for converting lysine into pipecolic acid^42^. Moreover, combining “Tropane, piperidine and pyridine alkaloid biosynthesis” pathway, we knew that there were 3 enzymes that play an important role in this two pathways, that is L-lysine oxidase (EC1.4.3.14), ∆^1^-piperideine-2-carboxylic reductase (EC1.5.1.21) and L-lysine-6- dehydrogenase (EC1.4.1.18). However, no clear gene was annotated to these enzymes, which confirmed previous studies^27^. In P6C pathway, we found L-2-aminoadipate 6-semialdehyde, is key intermediate compound in this route, according to L-lysine degradation pathway, it is catalyzed as a product of saccharopine by SDH, and 4 genes were annotated to this enzyme, which indicated that SDH played a very important role in swainsonine biosynthesis in *U. oxytropis*. Meanwhile, ∆^1^-piperideine-6-carboxylic acid (P6C) is also the important intermediates in P6C pathway^43^. Fujii *et al.*^45^ thought P5CR is responsible for the reduction of P6C into pipecolic acid^45^, our experimental results agreed, and we found that 2 genes were annotated to this enzyme, which indicated P5CR could be a key enzyme in swainsonine biosynthesis.

Polyketides, the ubiquitous products of secondary metabolism in microorganisms, are made by a process resembling fatty acid biosynthesis that allows the suppression of reduction or dehydration reactions at specific biosynthetic steps, giving rise to a wide range of often medically useful products^47^. The polyketide synthases (PKS) are a large class of natural products, which are produced by bacteria, actinomyces, fungi and plants. These natural products play an important role in anti-infection, anti-fungus, anti-tumor and immunologic suppression. In our studies, we also found 79 genes were annotated to PKS in *U. oxytropis* genome. Since 1-indolizinone is a ketone compound, we speculated that L-pipecolic acid could be changed into 1-indolizinone through PKS. But, so far we have no evidence to support this speculation, and this needs be confirmed by future research.

*Undifilum oxytropis* showed rich P450 family (77 genes annotated to cytochromes P450), which is involved in the biotransformation of drugs, the bioconversion of xenobiotics, the metabolism of chemical carcinogens, the biosynthesis of physiologically important compounds such as steroids, fatty acids, eicosanoids, the conversion of alkanes, terpenes, and aromatic compounds as well as the degradation of herbicides and insecticides. There is also a broad versatility of reactions catalysed by cytochromes P450 such as carbon hydroxylation and aromatic hydroxylation^48^. In biosynthesis of alkaloids derived from ornithine, lysine and nicotinate (KEGG map01064), we found that 1-indolizidinone was hydroxylated into swainsonine. As a result, we speculated that cytochromes P450 hydroxylase could also play a very important role in swainsonine biosynthesis pathway.

In conclusion, in *U. oxytropis* genome research, we clarified the sizes and characteristics of this fungus, and screened some genes that were annotated to key enzymes, which are theoretically involved in swainsonine biosynthesis by using whole genome shotgun sequencing strategy. In future, genes knockout of some key enzymes will be carried out and obtain mutant of *U. oxytropis*, which do not produce swainsonine, and on this basis we will gain a new locoweed species that do not contain swainsonine, this will fundamentally resolve locoweeds poisoning of animals and then implement the comprehensive utilization and management for locoweeds of grasslands in western China.

## Materials and Methods

### Strains and culture conditions

*Undifilum oxytropis* (OK3UNF) was isolated from *Oxytropis kansuensis*, a locoweed widely distributed in Qianlian County of Qinghai province in western China (38°3.249N, 100°13.660E), and deposited at the Animal Toxicology Institute of Northwest A&F University (Yangling, Shaanxi, China). The hyphal tipped culture of *U. oxytropis* was stored in a tube at 4 °C prior to transfer to fresh PDA (Potato Dextrose Agar) media and culture at room temperature. After 10-14 days growth, the mycelium was collected and preserved at 4 °C for genomic DNA isolation and extraction.

### DNA isolation, genome sequencing and assembly

Genomic DNA of *U. oxytropis* was isolated by an improved cetyl trimethylammonium bromide (CTAB) method^49^ and sequenced using a whole-genome shotgun strategy. All data were generated by paired-end sequencing of cloned inserts with insert size (500 bp) using an Illumina Hiseq2000 Sequencer at BGI-Shenzhen. After removing the low complexity, low quality, adapter and duplication contamination raw reads, the clean reads were assembled using the whole-genome *de novo* assembler SOAP *de novo*^29^,^50^.

### Genome Annotation

Protein coding gene models were predicted using *de novo* prediction tools SNAP^51^, GeneMarks^52^ and Augustus^53^ and homology based gene prediction tool Genewise^54^ with the default parameters. The homology-based and *de novo* gene sets were merged to form a comprehensive, non-redundant reference gene set by Glean^55^. The functional annotation of predicted gene models were mainly based on homology to known annotated genes; BLAST was the primary tool in our analyses. We aligned all protein models by BLASTP to SwissProt^56^, NR, and PHI^57^, P450^58^, CAZy^59^, and mapped them by function with GO^60^, COGs^61^,^62^ and KEGG pathways^63^,^64^,^65^. Since each gene mapped to different database sequences, there could be multiple aligned results meeting the cut-off, so the annotations of the sequences with the best score were chosen to be the annotation of the gene in *U. oxytropis*.

## Additional Information

**How to cite this article**: Lu, H. *et al.* The Genome of *Undifilum oxytropis* Provides Insights into Swainsonine Biosynthesis and Locoism. *Sci. Rep.* **6**, 30760; doi: 10.1038/srep30760 (2016).

## Supplementary Material

Supplementary Information

## Figures and Tables

**Table 1:** Description of genome assembly of *Undifilum oxytropis.*

Type	Genome
Scaffold Total number	6,367
Scaffold Total length (bp)	70,048,771
Scaffold N50 (bp)	33,191
Scaffold N90 (bp)	4,183
Scaffold Max length (bp)	345,265
Scaffold Min length (bp)	1,000
Contig Total number	9,757
Contig Total length (bp)	69,684,073
Contig N50 (bp)	24,851
Contig N90 (bp)	3,035
Contig Max length (bp)	293,689
Contig Min length (bp)	200
GC content (%)	40.37

**Table 2:** The putative genes involved in swainsonine biosynthesis.

Gene full name	Abbr.	Enzyme	KO	Putative gene
saccharopine dehydrogenase	SDH	EC:1.5.1.7	K00290	U_Oxytropis_10006494
		EC:1.5.1.9	K00292	U_Oxytropis_10009727
		EC:1.5.1.10	K00293	U_Oxytropis_10007601
				U_Oxytropis_10002513
				U_Oxytropis_10004318
saccharopine oxidase	FAP2	EC 1.5.3.1	K00301	U_Oxytropis_10007865
pyrroline-5-carboxylatereductase	P5CR	EC:1.5.1.2	K00286	U_Oxytropis_10009688
				U_Oxytropis_10002857
polyketide synthase	PKS	EC: 2.3.-.-		79 putative genes
cytochrome P450	P450	EC: 1.14.-.-		77 putative genes
