# Supplementary material for: The Genome of Undifilum oxytropis Provides Insights into Swainsonine Biosynthesis and Locoism
Source: Sci Rep. 2016 Aug 1;6:30760. doi: 10.1038/srep30760 (PMC4967851; doi:10.1038/srep30760)
Supplement: Supplementary Information [file srep30760-s1.doc]

**Supplementary information**

**"The Genome of *Undifilum oxytropis* Provides Insights into Swainsonine Biosynthesis and Locoism"**

**Hao Lu*, Haiyun Quan, Zhenhui Ren, Shuai Wang, Ruixu Xue, Baoyu Zhao***

**Figure S1. Distribution of qualities in per base of reads.**


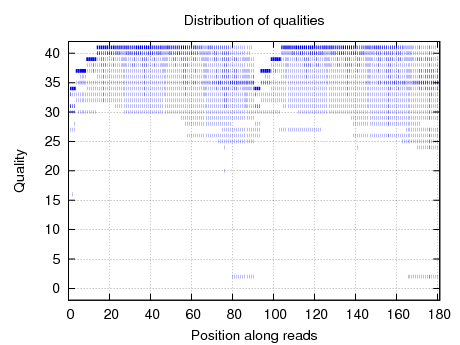


**Figure S2. Distribution of bases in each position of reads.**


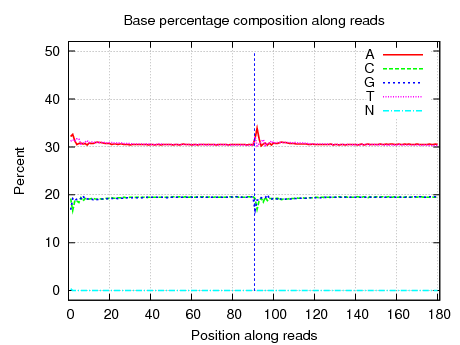


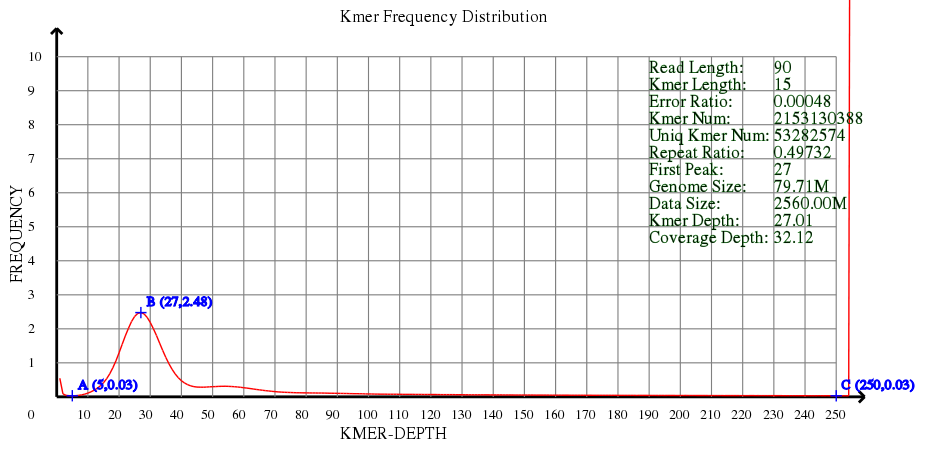
**Figure S3. 15-mer analysis chart**  Abscissa is depth, ordinate is proportion of the frequency account for total frequency under various depths. Without regard to error ratio of sequencing, heterozygosity and multiplicity of genome, 15-mer is chosen every base, and 15-mer distribution should obey poisson's distribution, but in actual data, K-mer number of low depth account for huge propotion due to exsiting senquencing error. Meanwhile, for certain genomes, heterozygosis will be appeared in a half of abscissa corresponding main peak because of certain heterozygosity, and certain multiplicity, repetition peak will be appeared in integral multiple of abscissa corresponding main peak.

**Fugure S4. The correlation analysis of GC contents and sequencing depth of *Udifilum Oxytropis*.** Absissa is GC content, ordinate is average sequencing depth. GC contents and average depth were calculated by using 500 bp window without duplication, sequencing data was analysed whether or not GC deviation according to this figure. Not serious data of GC deviation in sequencing process, the scatter diagram show a form of nearly poisson's distribution, a peak value will be showed nearby GC value of this genome, the greater distance to peak value, the lower coverage depth.


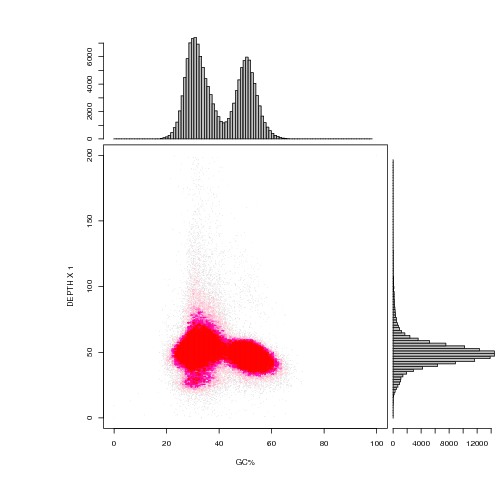


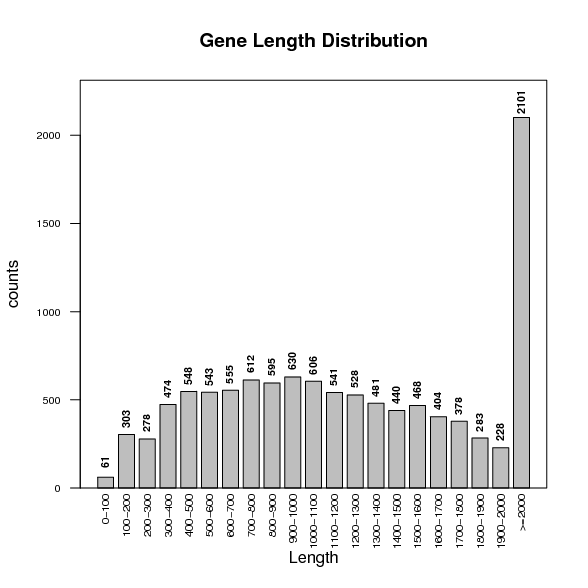
**Figure S5. Gene length distribution of *Undifilum oxytropis*.** The genome of *Undifilum oxytropis* was estimated and obtained gene length, 2101 genes are longer than 2000 bp in total genes.

**Table S1. The sequencing data of genome in *Undifilum Oxytropis*.** The genome of *Undifilum Oxytropis* was sequenced and produced 3,950 Mb data by using Illumina Hiseq2000 platform.

| **Sample Name** | **Insert Size (bp)** | **Reads length (bp)** | **Raw Data (Mb)** | **Adapter (%)** | **Duplication (%)** | **Total reads** | **Filtered reads (%)** | **Low quality filtered reads (%)** | **Clean Data (Mb)** |
| --- | --- | --- | --- | --- | --- | --- | --- | --- | --- |
| *Undifilum Oxytropis* | 500 | (90:90) | 4,214 | 0.02 | 1.02 | 46,821,396 | 6.26 | 2.87 | 3,950 |

**Table S2. The components of genome on *Undifilum Oxytropis.***

|  | ***Undifilum Oxytropis*** |
| --- | --- |
| **Genome Size** | 70,048,771 |
| **GC Content (%)** | 40.37 |
| **Number of Gene (#)** | 11,057 |
| **Number of Exons (#)** | 30,627 |
| **Number of CDS (#)** | 11,057 |
| **Number of Intron (#)** | 19,570 |
| **Length of Gene (bp)** | 18,400,467 |
| **Length of Exons (bp)** | 15,471,930 |
| **Length of CDS (bp)** | 15,471,930 |
| **Length of Intron (bp)** | 2,928,537 |
| **Average Length of Gene (bp)** | 1,664.15 |
| **Average Length of Exons (bp)** | 505.17 |
| **Average Length of CDS (bp)** | 1,399.29 |
| **Average Length of Intron (bp)** | 149.64 |
